# Detection of HER-3 with an AlGaN/GaN-Based Ion-Sensitive Heterostructure Field Effect Transistor Biosensor

**DOI:** 10.3390/mi14061186

**Published:** 2023-06-01

**Authors:** Fengge Wang, Honghui Liu, Yanyan Xu, Zhiwen Liang, Zhisheng Wu, Yang Liu, Baijun Zhang

**Affiliations:** 1 School of Electronics and Information Technology, Sun Yat-sen University, Guangzhou 510006, China; 2 State Key Laboratory of Optoelectronic Materials and Technologies, Sun Yat-sen University, Guangzhou 510275, China

**Keywords:** HER-3, biosensor, field effect transistor, AlGaN/GaN

## Abstract

Human epidermal growth factor receptor-3 (HER-3) plays a key role in the growth and metastasis of cancer cells. The detection of HER-3 is very important for early screening and treatment of cancer. The AlGaN/GaN-based ion-sensitive heterostructure field effect transistor (ISHFET) is sensitive to surface charges. This makes it a promising candidate for the detection of HER-3. In this paper, we developed a biosensor for the detection of HER-3 with AlGaN/GaN-based ISHFET. The AlGaN/GaN-based ISHFET biosensor exhibits a sensitivity of 0.53 ± 0.04 mA/dec in 0.01 M phosphate buffer saline (1× PBS) (pH = 7.4) solution with 4% bovine serum albumin (BSA) at a source and drain voltage of 2 V. The detection limit is 2 ng/mL. A higher sensitivity (2.20 ± 0.15 mA/dec) can be achieved in 1× PBS buffer solution at a source and drain voltage of 2 V. The AlGaN/GaN-based ISHFET biosensor can be used for micro-liter (5 μL) solution measurements and the measurement can be performed after incubation of 5 min.

## 1. Introduction

HER-3 is a member of the human epidermal growth factor receptor (HER) family. It plays a significant role in the growth of cells. The normal range of HER-3 in human blood is 60 pg/mL–2.55 ng/mL; however, it can rise to 12 ng/mL in cancer patients [1]. Cancer patients with high levels of HER-3 have poor treatment effects and significantly shortened survival times [2]. The latest research has found that reducing the concentration of HER-3 in these patients can effectively curb the growth and metastasis of cancer cells [3]. As an important biomarker of cancer, the detection of HER-3 is conducive to early and timely screening and treatment of cancer. Many techniques such as fluorescence [4], immunohistochemistry [5,6], enzyme linked immuno assay (ELISA) [7,8], Northern blot [9], and Southern blot [10] are currently widely used in the detection of HER-3. These detection methods are time-consuming and expensive. Furthermore, they require large sample volumes and professionals. The field effect transistor (FET) has the advantages of small size, low cost, no label, and has been applied to the detection of HER-3 [11,12,13,14,15]. Because the moving charges of biological samples can shield the target charges, the sensitivity of the sensor is greatly reduced. High sensitivity can be obtained in the solution with low ion concentration [16,17]. The use of electrochemical impedance spectroscopy and nano materials (carbon nanotubes, graphene) is conducive to overcoming the charge shielding effect and improving the sensitivity of biosensors [11,12,13,14,15]. The distance between the reference electrode and the sensitive area, and the voltage of the reference electrode will also affect the sensitivity of the biosensor [18,19]. Recently, AlGaN and GaN materials have been favored by people due to their advantages such as surface sensitivity, high mobility, good biocompatibility, and low power consumption [20]. The AlGaN/GaN ion-sensitive heterostructure field effect transistor (ISHFET) is very sensitive to surface charges due to the existence of two-dimensional electron gas (2DEG) and this makes it ideal for detecting biomarkers [21]. Compared with other semiconductor materials, the limited Fermi level pinning effect makes these materials exhibit excellent sensing properties [22]. The use of silicon substrate is beneficial to integration and cost reduction [23]. In this paper, an AlGaN/GaN-based ISHFET biosensor was developed and used to detect HER-3. The AlGaN/GaN-based ISHFET biosensor shows a sensitivity of 0.53 ± 0.04 mA/dec in 1× PBS solution with 4% BSA at a source and drain voltage of 2 V. The measurements can be achieved in 5 min with 5 μL sample.

## 2. Device Fabrication

**Figure 1:**
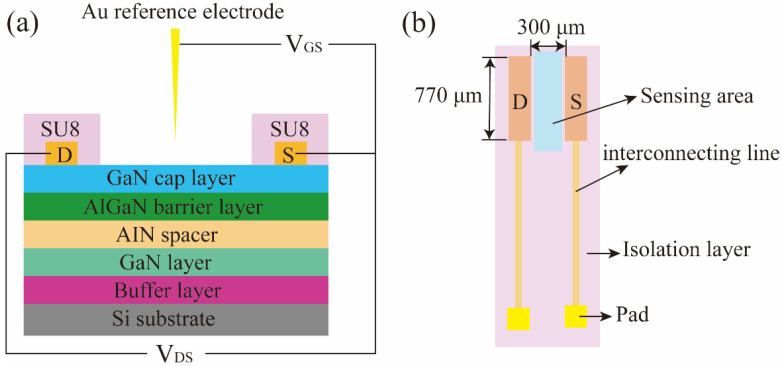
(**a**) The cross-sectional view of the AlGaN/GaN-based ISHFET. (**b**) The top view of the AlGaN/GaN-based ISHFET. (D: Drain, S: Source).

The AlGaN/GaN heterostructure was grown by metal–organic chemical vapor deposition (MOCVD). The epilayer included a 2 nm GaN cap layer, a 25 nm AlGaN barrier layer, an AIN spacer layer, an i-GaN channel layer, and a buffer layer grown on silicon substrate. Firstly, a depth of 110 nm mesa isolation was achieved by an inductively coupled plasma reactive ion etching (ICP-RIE) system (Wavetest, AST Cirie-200S) with Cl_2_/BCl_3_ mixed gases. Then, the source and drain electrodes were formed by depositing Ti/Al/Ni/Au (15/80/20/60 nm) with an electron beam evaporation device (Wavetest, ULVAC KIKO VPC-1100). The device was subsequently annealed at 850 °C in N_2_ for 30 s. Afterwards, a multiple metal structure of Ti/Au (5 nm/100 nm) was deposited via electron beam evaporation to form interconnecting metal lines and test pads. Lastly, an SU8 layer was used to cover the AlGaN/GaN based ISHFET biosensor except for the sensing area between source and drain and the testing pads. The cross-sectional and top views of the ISHFET are shown in Figure 1a,b. The gate width and length were 770 μm and 300 μm, respectively.

## 3. Surface Functionalization

**Figure 2:**
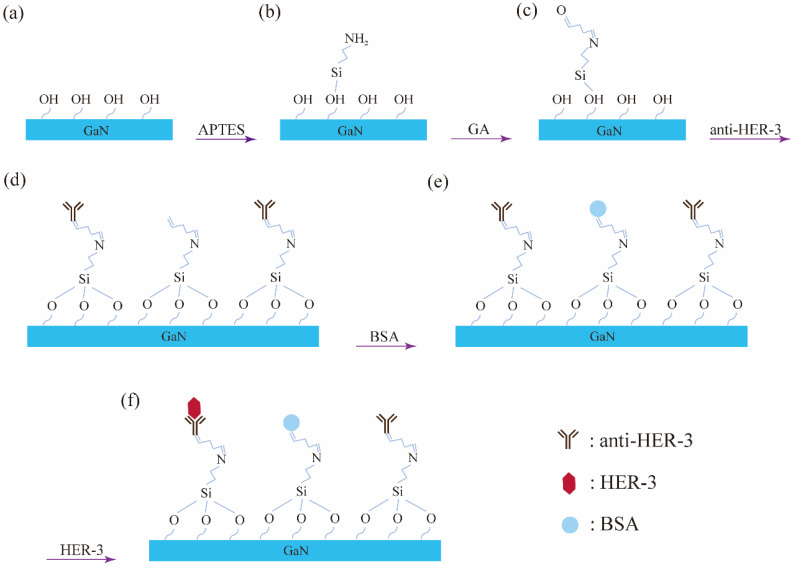
(**a**) Bare GaN surface. (**b**) GaN surface was modified by -NH_2_. (**c**) GaN surface was modified by -CHO. (**d**) anti-HER-3 was immobilized on GaN surface. (**e**) BSA was immobilized on GaN surface to avoid nonspecific binding of HER-3. (**f**) Binding of HER-3 with anti-HER-3.

**Figure 3:**
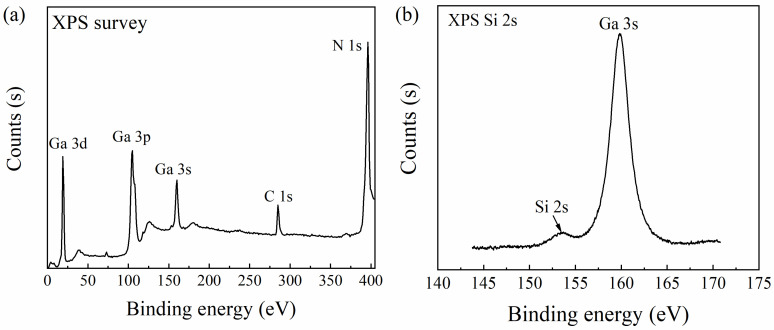
(**a**) XPS spectrum, (**b**) Si 2 s high-resolution XPS spectrum after APTES functionalization.

**Figure 4:**
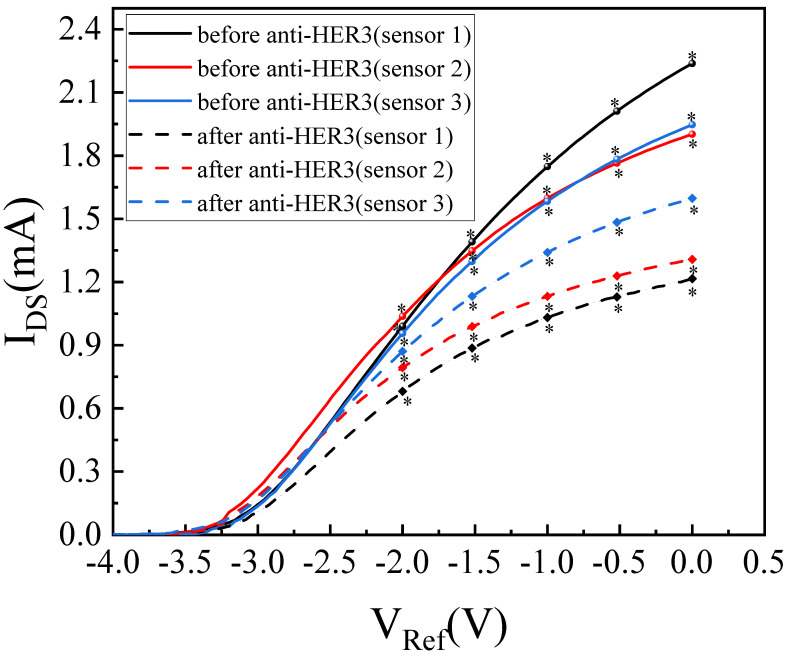
Transfer characteristics of the biosensor before and after anti-HER-3 incubation. * states for *p* values of <0.05.

Firstly, the encapsulated AlGaN/GaN-based ISHFET was immersed in a 3-aminopropyltrieth-oxysilane (APTES) solution (APTES 15% in ethanol) for 12 h. There were native oxides on the sensing area and the hydroxyl groups reacted with APTES. After immersing in APTES solution, the sensing area was modified by the amino group(-NH_2_), as shown in Figure 2b. After that, the AlGaN/GaN-based ISHFET was immersed in a glutaraldehyde (GA) solution (GA 25% in pure water) for 2 h. During this process, GA reacted with -NH_2_ and yielded the aldehyde group (-CHO), as shown in Figure 2c. To detect HER-3, the anti-HER-3-HER-3 ligand-receptor system was adopted. As shown in Figure 2d, the anti-HER-3 was then immobilized by forming an imine linkage with the aldehyde group by exposing in 0.01 M phosphate buffer saline (1× PBS) (pH = 7.4) solution of anti-HER-3 for 2 h. Finally, the sensing area was exposed in 1% bovine serum albumin (BSA) solution. The nonspecific binding of HER-3 was avoided, as shown in Figure 2e [24]. All operations were carried out at room temperature. The device was thoroughly washed with deionized water and dried in nitrogen flow when changing solutions. Figure 2a–e illustrates the functionalization steps of the sensing area. Figure 2f shows the binding of HER-3 with anti-HER-3. The immobilization of APTES on GaN surface was verified by X-ray photoelectron spectroscopy (XPS). The XPS spectrum and Si 2s high-resolution spectrum after APTES functionalization are shown in Figure 3a,b. The Si 2s emission with binding energy of 153.43 eV in Figure 3b indicates the immobilization. The surface functionalization can also be confirmed by testing the transfer curves before and after anti-HER-3 incubation. The transfer curves were tested at room temperature using a semiconductor device analyzer (Aglient B 1500A, Santa Rosa, CA, USA). The voltage of source and drain electrodes was set at 0.5 V. The curves tested with 1× PBS buffer solution are shown in Figure 4. Obviously, the current changed after the device was exposed in anti-HER-3 solution (one-way ANOVA, *p* < 0.05, n = 3). The change in current originated from the covalent coupling of anti-HER-3. The changes in surface charges caused by anti-HER-3 influenced the 2DEG, which decreased the conductance of the AlGaN/GaN-based ISHFET biosensor [21].

## 4. Detection of HER-3 in 1× PBS Buffer Solution with 4% BSA

**Figure 5:**
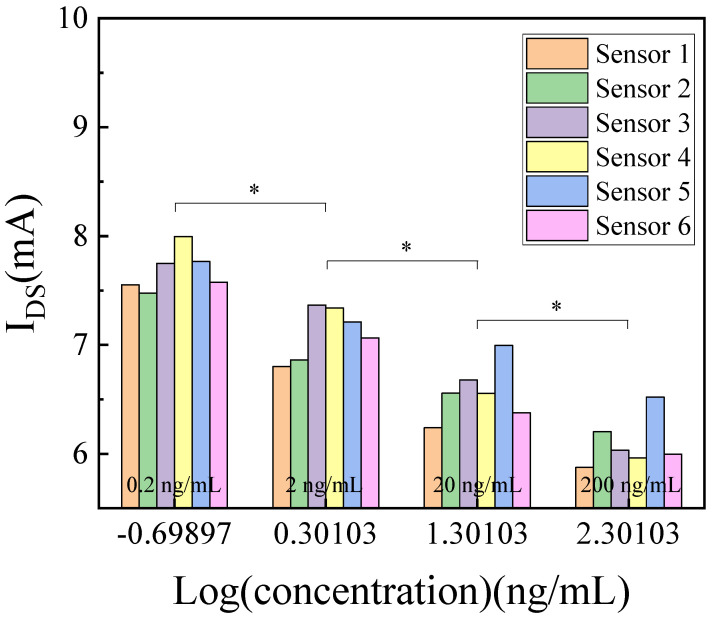
HER-3 detection of the AlGaN/GaN-based ISHFET biosensor in 1× PBS buffer solution with 4% BSA. * states for *p* values of <0.05.

To evaluate the performance of the AlGaN/GaN-based ISHFET biosensor, different concentrations of HER-3 were prepared and measured. HER-3 was diluted to a series of concentrations in 1× PBS buffer solution with 4% BSA. Prior to electrical testing, HER-3 solutions (5 µL) were introduced on the sensing area and incubated in a humid environment at room temperature for 5 min. The source and drain currents (I_DS_) of the AlGaN/GaN-based ISHFET biosensor were recorded by the semi-conductor device analyzer at room temperature. The voltage of source and drain electrodes was set at 2 V, and the voltage of Au reference electrode was set at 1 V. Figure 5 shows the current responses of the ISHFET biosensor for different HER-3 solutions. The currents of 0.2 ng/mL, 2 ng/mL, 20 ng/mL and 200 ng/mL were 7.69 ± 0.08 mA, 7.11 ± 0.10 mA, 6.57 ± 0.11 mA, 6.10 ± 0.09 mA, respectively (*p* < 0.05, n = 6). The currents decreased as HER-3 increased. The binding of HER-3 influenced the concentration of the 2DEG in the AlGaN/GaN interface and finally resulted in a decrease in current for the devices. A sensitivity of 0.53 ± 0.04 mA/dec was achieved using the AlGaN/GaN-based ISHFET biosensor. Table 1 shows a comparison of our work with previously reported work. 

## 5. Detection of HER-3 in 1× PBS Buffer Solution

**Figure 6:**
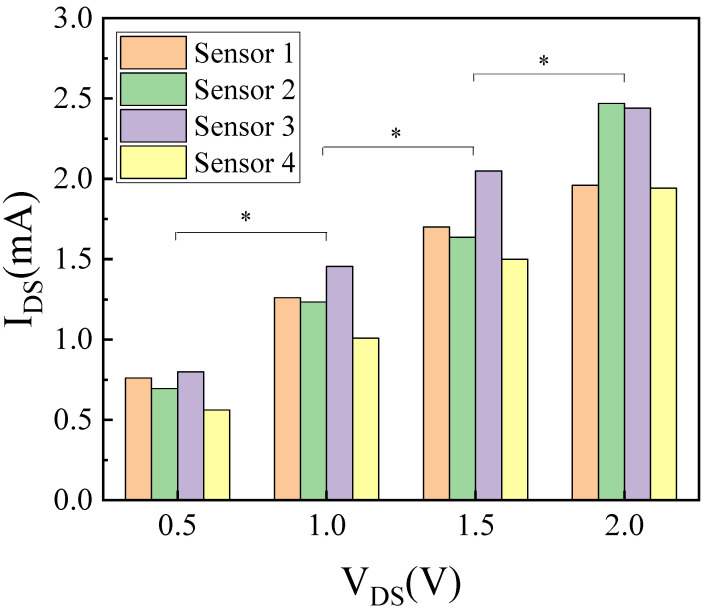
HER-3 detection of the AlGaN/GaN-based ISHFET biosensor in 1× PBS buffer solution. * states for *p* values of < 0.05.

We also tested the performance of the AlGaN/GaN-based ISHFET biosensor in 1× PBS buffer solution under a different source and drain voltage. Herein, HER-3 was diluted to a series of concentrations in 1× PBS buffer solution. The voltage of Au reference electrode was set at 1 V, and the source and drain voltage were set at 0.5 V, 1 V, 1.5 V, and 2 V. Prior to electrical testing, HER-3 solutions (5 µL) were introduced on the sensing area and incubated in a humid environment at room temperature for 5 min. Figure 6 shows the sensitivity of the biosensor at a different source and drain voltage in 1× PBS buffer solution. The sensitivities of the biosensor were 0.70 ± 0.05 mA/dec, 1.24 ± 0.09 mA/dec, 1.72 ± 0.12 mA/dec, 2.20 ± 0.15 mA/dec at a source and drain voltage of 0.5 V, 1 V, 1.5 V, and 2 V, respectively (*p* < 0.05, n = 4). A higher sensitivity can be achieved at a source and drain voltage of 2 V. Due to the large leakage current, the ISHFET biosensor cannot work well when the voltage of source and drain was set at 2.5 V.

## 6. Selectivity and Storage Stability of the Biosensor

In order to verify the selectivity of the biosensor, some interfering proteins (2 ng/mL streptavidin) were added to HER-3 solutions (2 ng/mL) in 1× PBS buffer solution with 4% BSA. The electrical signals of the biosensor were tested when there were no interference proteins and when there were interference proteins. The test results showed that the interfering proteins had no effect on the biosensor. The storage stability of the biosensor was also investigated. The biosensor was stored in a refrigerator at 4 °C. The test solutions were 2 ng/mL HER-3 solutions in 1× PBS buffer solution with 4% BSA. The electrical signal of the biosensor was 97.5% of the initial signal after 5 days, demonstrating that the biosensor has an acceptable stability.

## 7. Conclusions

In this paper, we fabricated a HER-3 biosensor using the AlGaN/GaN-based ISHFET. The sensing area was functionalized using APTES and GA to immobilize a probe (anti-HER-3). The chemical groups after APTES functionalization were confirmed by XPS. Our data show that the AlGaN/GaN based ISHFET biosensor can detect HER-3 in 1× PBS buffer solution with 4% BSA, and the sensitivity is about 0.53 ± 0.04 mA/dec at a source and drain voltage of 2 V. A higher sensitivity (2.20 ± 0.15 mA/dec) can be achieved in 1× PBS buffer solution without BSA at a source and drain voltage of 2 V. Since the miniaturization without sample dilution, the detection can be achieved with a small sample volume (<5 μL) and much less time (5 min).

## Figures and Tables

**Table 1:** Comparison of our work with previously reported works.

Technique of Detection	The Type of Immunosensor	Immobilizing Biomolecules	Analyte	Linear Range	LOD	Ref.
electrochemical impedance spectroscopy	electrochemistry	anti-HER3	HER3	0.2–1.4 pg/mL	0.2 pg/mL	[11]
electrochemical impedance spectroscopy	electrochemistry	anti-HER3	HER3	2–14 fg/mL	2 fg/mL	[12]
single frequency impedance	electrochemistry	anti-HER3	HER3	40–200 fg/mL	40 fg/ml	[13]
electrochemical impedance spectroscopy	electrochemistry	anti-HER3	HER3	0.4–2.4 pg/mL	0.4 pg/mL	[14]
current–gate voltage measurements	electrochemistry	anti-HER3	HER3	300 fg/mL–300 ng/mL	300 fg/mL	[15]
current–gate voltage measurements	electrochemistry	anti-HER3	HER3	2–200 ng/mL	2 ng/mL	our work

## Data Availability

The data that support the finding of this study are available from the corresponding author upon reasonable request.

## References

[1] Lédel F., Hallström M., Ragnhammar P., Öhrling K., Edler D. (2014). HER-3 expression in patients with primary colorectal cancer and corresponding lymph node metastases related to clinical outcome. Eur. J. Cancer.

[2] Tanner B., Hasenclever D., Stern K., Schormann W., Bezler M., Hermes M., Brulport M., Bauer A., Schiffer I.B., Gebhard S. (2006). ErbB-3 predicts survival in ovarian cancer. J. Clin. Oncol..

[3] Kol A., van Scheltinga A.G.T., Timmer-Bosscha H., Lamberts L.E., Bensch F., de Vries E.G., Schröder C.P. (2014). HER3, serious partner in crime: Therapeutic approaches and potential biomarkers for effect of HER3-targeting. Pharmacol. Ther..

[4] Offterdinger M., Schöfer C., Weipoltshammer K., Grunt T.W. (2002). C-erbB-3: A nuclear protein in mammary epithelial cells. J. Cell Biol..

[5] Hilbe W., Dirnhofer S., Oberwasserlechner F., Eisterer W., Ammann K., Schmid T. (2003). Immunohistochemical typing of non-small cell lung cancer on cryostat sections: Correlation with clinical parameters and prognosis. J. Clin. Pathol..

[6] Valsamo K.A., Allison W.W., Giltnane J.M., Siddiqui S., Liceaga C., Gustavson M., Syrigos K.N., Reiter J.L., Rimm D.L. (2010). Analytic Variability in Immunohistochemistry Biomarker Studies. Cancer Epidemiol. Biomarkers Prev..

[7] Aurisicchio L., Marra E., Luberto L., Carlomosti F., De Vitis C., Noto A., Gunes Z., Roscilli G., Mesiti G., Mancini R. (2012). Novel anti-ErbB3 monoclonal antibodies show therapeutic efficacy in xenografted and spontaneous mouse tumors. J. Cell. Physiol..

[8] Hsieh S.Y., He J.R., Yu M.C., Lee W.C., Chen T.C., Lo S.J., Bera R., Sung C.M., Chiu C.T. (2011). Secreted ERBB3 Isoforms Are Serum Markers for Early Hepatoma in Patients with Chronic Hepatitis and Cirrhosis. J. Proteome Res..

[9] Lemoine N.R., Barnes D.M., Hollywood D.P., Hughes C.M., Smith P., Dublin E., Prigent S.A., Gullick W.J., Hurst H.C. (1992). Expression of the ERBB3 gene product in breast cancer. Br. J. Cancer.

[10] Kraus M.H., Issing W., Miki T., Popescu N.C., Aaronson S.A. (1989). Isolation and characterization of ERBB3, a third member of the ERBB/epidermal growth factor receptor family: Evidence for overexpression in a subset of human mammary tumors. Proc. Natl. Acad. Sci. USA.

[11] Canbaz M.Ç., Şimşek Ç.S., Sezgintürk M.K. (2014). Electrochemical biosensor based on self-assembled monolayers modified with gold nanoparticles for detection of HER-3. Anal. Chim. Acta..

[12] Asav E., Sezginturk M.K. (2014). A novel impedimetric disposable immunosensor for rapid detection of a potential cancer biomarker. Int. J. Biol. Macromol..

[13] Canbaz M.Ç., Sezgintürk M.K. (2014). Fabrication of a highly sensitive disposable immunosensor based on indium tin oxide substrates for cancer biomarker detection. Anal. Biochem..

[14] Sonuç M.N., Sezgintürk M.K. (2014). Ultrasensitive electrochemical detection of cancer associated biomarker HER3 based on anti HER3 biosensor. Talanta.

[15] Rajesh, Gao Z., Vishnubhotla R., Ducos P., Serrano M.D., Ping J., Robinson M.K., Johnson A.T.C. (2016). Genetically engineered antibody functionalized Platinum nanoparticles modified CVD-graphene nanohybrid transistor for the detection of breast cancer biomarker, HER3. Adv. Mater. Interfaces.

[16] Stern E., Wagner R., Sigworth F.J., Breaker R., Fahmy T.M., Reed M.A. (2007). Importance of the Debye Screening Length on Nanowire Field Effect Transistor Sensors. Nano Lett..

[17] Kesler V., Murmann B., Soh H.T. (2020). Going beyond the Debye Length:Overcoming Charge Screening Limitations in Next Generation Bioelectronic Sensors. ACS Nano.

[18] Wu C.-R., Wang S.-L., Chen P.-H., Wang Y.-L., Wang Y.-R., Chen J.-C. (2021). Demonstration of the enhancement of gate bias and ionic strength in electricdouble-layer field-effect-transistor biosensors. Sens. Actuators B Chem..

[19] Tai T.-Y., Sinha A., Sarangadharan I., Pulikkathodi A.K., Wang S.-L., Lee G.-Y., Chyi J.-I., Shiesh S.-C., Lee G.-B., Wang Y.-L. (2019). Design and demonstration of tunable amplified sensitivity of algan/gan high electron mobility transistor (hemt)-based biosensors in human serum. Anal. Chem..

[20] Mounika B., Ajayan J., Bhattacharya S., Nirmal D. (2022). Recent developments in materials, architectures and processing of AlGaN/GaN HEMTs for future RF and power electronic applications: A critical review. Micro Nanostructures.

[21] Steinhoff G., Hermann M., Schaff W.J. (2003). pH response of GaN surfaces and its application for pH-sensitive field-effect transistors. Appl. Phys. Lett..

[22] Ajayan J., Nirmal D., Ramesh R., Bhattacharya S., Tayal S., Joseph L.L., Thoutam L.R., Ajitha D. (2021). A critical review of AlGaN/GaN-heterostructure based Schottky diode/HEMT hydrogen (H2) sensors for aerospace and industrial applications. Measurement.

[23] Ajayan J., Nirmal D., Mohankumar P., Mounika B., Bhattacharya S., Tayal S., Fletcher A.S.A. (2022). Challenges in material processing and reliability issues in AlGaN/GaN HEMTs on silicon wafers for future RF power electronics & switching applications: A critical review. Mater. Sci. Semicond. Process..

[24] Vashist S.K., Lam E., Hrapovic S., Male K.B., Luong J.H.T. (2014). Immobilization of antibodies and enzymes on 3-aminopropyltriethoxysilane functionalized bioanalytical platforms for biosensors and diagnostics. Chem. Rev..

